# Single-stage repair of aortic arch hypoplasia and ventricular septal defect in a low-weight infant: a case report from a resource-limited center

**DOI:** 10.47487/apcyccv.v6i3.498

**Published:** 2025-09-24

**Authors:** Jesús García Pinzás, Cristhian J Cueva Alvarez, Martin Crosby Barabino

**Affiliations:** 1 Instituto Nacional de Salud del Niño de Breña, Lima, Perú. Instituto Nacional de Salud del Niño de Breña Lima Perú; 2 Universidad de San Martín de Porres, Lima, Perú. Universidad de San Martín de Porres Universidad de San Martín de Porres Lima Peru; 3 Universidad Continental, Lima, Perú. Universidad Continental Lima Perú

**Keywords:** Aortic Coarctation, Heart Defects, Congenital, Cerebrovascular Circulation, Coartación aórtica, Cardiopatías Congénitas, Circulación Cerebrovascular

## Abstract

Aortic arch hypoplasia associated with ventricular septal defect (VSD) is a life-threatening congenital condition that demands early intervention. In low-resource settings, the lack of advanced tools complicates the safe use of selective antegrade cerebral perfusion (SACP) and innovative repair techniques such as interdigitating patch reconstruction. We describe the case of a newborn with severe aortic arch hypoplasia and a large perimembranous VSD who underwent successful one-stage surgical correction. The repair included aortic arch reconstruction using an interdigitating bovine pericardial patch and VSD closure under SACP delivered via direct brachiocephalic trunk cannulation. Despite the challenges of limited monitoring and equipment, the infant had an excellent clinical outcome. This case highlights the feasibility of adapting high-complexity cardiac techniques in resource-limited environments when surgical fundamentals and teamwork are prioritized. With strategic planning, multidisciplinary coordination, and adherence to evidence-based principles, successful single-stage repair of complex congenital heart disease is achievable even in under-resourced settings.

## RESUMEN

La hipoplasia del arco aórtico asociada a comunicación interventricular (CIV) constituye una anomalía congénita severa que requiere una intervención quirúrgica temprana. La ausencia de modalidades avanzadas de perfusión y de tecnologías de neuromonitoreo plantea retos para la implementación segura de la perfusión cerebral anterógrada selectiva (PCAS) y la adopción de estrategias quirúrgicas como la reconstrucción con parche interdigitado. Presentamos el caso de un recién nacido con hipoplasia severa del arco aórtico y una CIV perimembranosa amplia, quien fue sometido con éxito a corrección quirúrgica en un solo tiempo. La estrategia operatoria incluyó la reconstrucción del arco aórtico mediante un parche interdigitado de pericardio bovino y el cierre de la CIV, realizados bajo PCAS administrada mediante canulación directa del tronco braquiocefálico. Demostramos la viabilidad de adaptar técnicas complejas en cirugía cardíaca a entornos con recursos limitados, siempre que se prioricen los fundamentos quirúrgicos, el trabajo en equipo y la coordinación multidisciplinaria.

## Introduction

Aortic arch hypoplasia combined with a ventricular septal defect (VSD) represents a challenging entity in neonatal cardiac surgery. ^1^ In high-income countries, one-stage neonatal repair using selective antegrade cerebral perfusion (SACP) and patch aortoplasty has become the standard for optimizing outcomes. ^2^^,^^3^ The presence of a hypoplastic aortic arch, especially in combination with a VSD, significantly increases the complexity of surgical repair due to altered hemodynamics, the risk of recoarctation, and the need for cerebral and visceral organ protection during cardiopulmonary bypass. ^3^^-^^5^

Over the past two decades, selective antegrade cerebral perfusion (SACP) has become the preferred neuroprotective strategy during neonatal aortic arch reconstruction, replacing deep hypothermic circulatory arrest (DHCA) in many high-volume centers. ^6^^-^^8^ SACP allows continuous oxygenation of the brain while reducing the risks of neurological injury and systemic inflammation. Various cannulation techniques-including direct brachiocephalic trunk artery cannulation-have been shown to be safe and effective even in neonates. ^5^^,^^6^

Nevertheless, these strategies are highly dependent on advanced perfusion equipment, specialized monitoring (e.g., near-infrared spectroscopy), and well-trained perfusion teams. In low-resource settings, these components are often limited or absent. Consequently, the adaptation of neuroprotective strategies such as SACP must be carefully tailored to institutional capacity while maintaining surgical safety. ^2^^,^^9^

The surgery was carried out at a tertiary cardiac surgery unit with significant resource limitations, including the absence of advanced cardiac computed tomography (CT), nitric oxide therapy, and extracorporeal membrane oxygenation (ECMO), as well as only three pediatric intensive care unit beds. At the time, no vascular grafts were available. We report the case of a full-term neonate with hypoplastic aortic arch and ventricular septal defect who underwent single-stage repair using selective antegrade cerebral perfusion through direct innominate artery cannulation.

## Case report

A full-term male infant was delivered at 38 weeks of gestation with a birth weight of 2,200 g. Soon after birth, he developed respiratory distress with persistent hypoxemia (oxygen saturation around 70% in room air) and signs of congestive heart failure, requiring supplemental oxygen and diuretic therapy.

**Figure 1:**
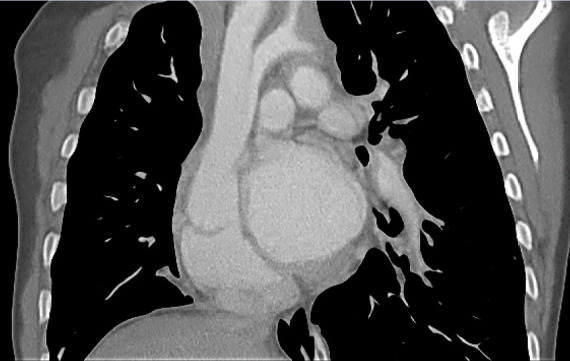
Computed tomography with coronal view: A severe hypoplasia of the distal aortic arch is observed. The persistent ductus arteriosus is visualized as a vascular connection between the left pulmonary artery and the descending aorta.

**Figure 2:**
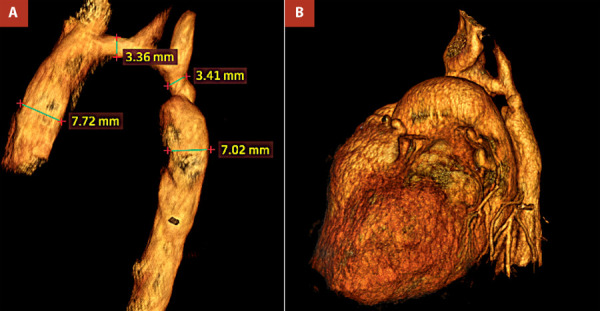
A. Three-dimensional volume-rendered computed tomography (CT) reconstruction of the thoracic great vessels demonstrates severe hypoplasia of the aortic arch, with significant narrowing of the proximal and distal segments measuring 3.36 mm and 3.41 mm, respectively. A markedly enlarged patent ductus arteriosus (PDA), measuring 7.02 mm, connects the pulmonary artery to the descending aorta. Postductal dilation of 7.72 mm is observed, consistent with retrograde systemic perfusion via the PDA. B. The 3D volume-rendered CT angiogram reveals a markedly hypoplastic transverse aortic arch, with critical narrowing at the level of the PDA, severely compromising the continuity of systemic flow through the arch.

Echocardiography revealed a large perimembranous ventricular septal defect with a left-to-right shunt, severe transverse aortic arch hypoplasia with critical narrowing at the isthmus, and a large patent ductus arteriosus (PDA) carrying retrograde flow to the descending aorta-indicating ductus-dependent systemic perfusion. CT confirmed the diagnosis, showing marked underdevelopment of the transverse arch and isthmus, with distal systemic flow sustained via the PDA (Figure 1-2).

Medical stabilization was achieved with prostaglandin E1 infusion to maintain ductal patency. At one month of age, with a weight of 2,920 g, the patient was taken to the operating room for urgent single-stage repair. Through a median sternotomy, surgical inspection confirmed severe arch hypoplasia, 4 mm at its narrowest point, a large perimembranous VSD (10 mm), and an 8 mm PDA. Cardiopulmonary bypass was established under moderate systemic hypothermia 30 °C. SACP was delivered via direct brachiocephalic trunk cannulation at 25 °C and maintained for 60 minutes during circulatory arrest to allow safe reconstruction of the arch.

**Figure 3:**
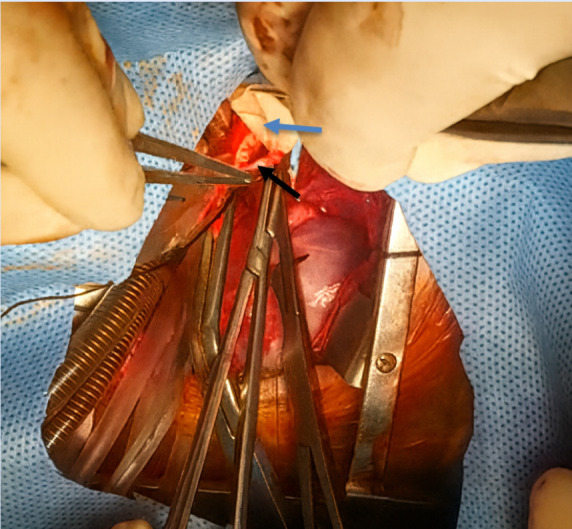
Intraoperative photograph during aortic arch reconstruction in a neonate with severe arch hypoplasia. The anterior bovine pericardial patch is being sutured using the interdigitating technique to augment the hypoplastic aortic arch. Black arrow: Aortic arch. Blue arrow: Pericardial patch.

The hypoplastic arch was augmented using an anterior bovine pericardial patch with the interdigitating technique, and the PDA was ligated. The VSD was closed with a bovine pericardial patch (Figure 3). Due to myocardial edema, the sternum was left open and closed 24 hours later without infectious complications, with sinus rhythm preserved throughout.

The postoperative course included transient low cardiac output and supraventricular tachycardia, both of which resolved with medical therapy. The patient was extubated after 72 hours and transferred out of the intensive care unit on postoperative day 9. At discharge, the neurological examination was normal, with preserved reflexes, spontaneous limb movements, and visual tracking.

By the last follow-up in July 2025, six months after surgery, he demonstrated normal psychomotor development, no neurological deficits, and weighed 8 kg.

## Discussion

The coexistence of severe aortic arch hypoplasia and a large perimembranous VSD in a neonate represents a critical surgical challenge. Early complete repair is preferred to avoid circulatory instability, minimize pulmonary overcirculation, and prevent irreversible end-organ dysfunction. ^1^^,^^2^ The current case highlights the feasibility of performing such complex reconstruction in a resource-limited setting, utilizing SACP and interdigitating arch augmentation, strategies typically reserved for high-complexity centers.

**Figure 4:**
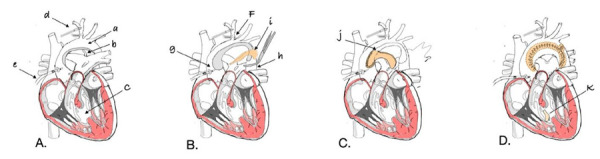
Schematic representation of the surgical technique. **(A)** Depicts the hypoplastic aortic arch (a), persistent ductus arteriosus (b), and ventricular septal defect (c). Arterial cannulation (d) is performed through the brachiocephalic artery, and a coronary perfusion cannula (e) is inserted into the ascending aorta. **(B)** At 28°C, the brachiocephalic artery (f), ascending aorta (g), and descending aorta (h) are clamped to enable continuous selective cerebral and myocardial perfusion while performing aortic arch reconstruction with a bovine pericardial patch (i). **(C)** Following aortoplasty, cardiac arrest is induced, and the proximal anastomosis is performed with the open ascending aorta (j). **(D)** Finally, systemic flow is resumed, rewarming is initiated, and the ventricular septal defect is closed with a heterologous patch (k). Illustration created by the author.

In recent decades, one-stage repair with arch reconstruction and VSD closure has become standard for neonates with complex aortic arch obstruction. ^3^^,^^4^ Several techniques for arch reconstruction are described, including extended end-to-end, end-to-side, and patch aortoplasty. Among these, the interdigitating technique has demonstrated superior anatomical alignment and reduced rates of restenosis or reintervention. ^10^ In a series by Winder *et al*., the interdigitating method decreased reintervention rates from 31% to 13%, even in Norwood patients. ^10^ Our decision to employ an anterior bovine pericardial patch with interdigitating suture was guided by the arch geometry and the desire to minimize tension and maintain laminar flow across the repair site. ^10^^,^^11^ A schematic diagram of the surgical technique, detailing the placement of the patch and interdigitating suture, is presented to illustrate the procedure **(**Figure 4**)**.

Cerebral protection is paramount during arch reconstruction. Historically, DHCA was widely used but has been associated with an increased risk of neurologic injury and delayed neurodevelopment. ^6^^,^^7^ SACP is now favored in many centers due to its superior metabolic and neurological profile. ^8^^,^^9^^,^^12^^-^^15^ Studies such as those by Soynov et al. and Kulyabin et al. support the use of SACP in neonates, showing preserved end-organ function, stable lactate clearance, and lower biomarkers of neurologic injury. ^3^^,^^15^ Our patient underwent SACP via direct brachiocephalic artery cannulation, a technique shown to be safe and reproducible even in neonates with small vessel diameters. ^5^ Despite a 60-minute arrest period, the patient had no postoperative neurologic deficits, highlighting the protective effect of SACP, even without neuromonitoring.

This is particularly relevant in low-resource environments, where near-infrared spectroscopy (NIRS), continuous electroencephalography, or dedicated perfusion teams may be unavailable. Studies like those of Fraser and Andropoulos emphasize the critical balance between technique and available infrastructure. ^13^ Our approach relied on meticulous perfusion control and team coordination, using SACP at moderate hypothermia (25 °C) to maintain cerebral flow while minimizing systemic ischemia.

Moreover, anatomical definitions of arch hypoplasia vary. Parikh et al. proposed using arch diameter ratios specifically distal transverse arch diameter/carotid artery diameter (DT/CA < 0.9), as an indicator of the surgical threshold for median sternotomy and full arch repair. ^16^ Our patient’s transverse arch measured 4 mm, consistent with severe hypoplasia. ^16^ Additional data from Li et al. and Margarint et al. support early intervention in patients with borderline arch geometry to prevent systemic hypertension and long-term recoarctation. ^11^^,^^17^

Despite transient postoperative supraventricular tachycardia and low cardiac output, our patient stabilized with medical therapy. Notably, sternal closure was delayed for 24 hours, a strategy often adopted to reduce tamponade risk in neonates with myocardial edema. ^8^ The absence of infectious complications and the preserved neurological status at discharge support the overall safety of our approach.

Finally, this case demonstrates that advanced surgical techniques can be successfully applied in resource-constrained settings, provided that the core principles of congenital heart surgery are respected and adapted. As echoed by Kozyrev et al. and Onalan et al., even in less equipped centers, structured protocols, careful perfusion planning, and judicious technique selection can yield outcomes comparable to those of high-volume institutions. ^7^^-^^9^

In conclusion this case demonstrates the feasibility of one-stage aortic arch and VSD repair in low-weight infants within resource-limited centers. The use of an interdigitating patch and direct SACP provided anatomical and neurological protection, even in the absence of advanced support tools. Such results reinforce the viability of evidence-based approaches in diverse healthcare contexts. ^11^^,^^16^

Our experience reinforces that meticulous surgical planning, adherence to cerebral protection principles, and adaptation of high-complexity techniques are essential to improving outcomes for neonates with complex congenital heart disease even in centers with limited resources. This case highlights a replicable model for other institutions facing similar constraints and contributes to the growing global discourse on equity and excellence in pediatric cardiac surgery.
